# Estimation of maximum body size in fossil species: A case study using *Tyrannosaurus rex*

**DOI:** 10.1002/ece3.11658

**Published:** 2024-07-24

**Authors:** Jordan C. Mallon, David W. E. Hone

**Affiliations:** ^1^ Beaty Centre for Species Discovery and Palaeobiology Section, Canadian Museum of Nature Ottawa Ontario Canada; ^2^ Department of Earth Sciences Carleton University, 2115 Herzberg Laboratories Ottawa Ontario Canada; ^3^ School of Biological and Behavioural Sciences Queen Mary University of London London UK

**Keywords:** body mass, dinosaurs, ontogeny, palaeoecology, sexual size dimorphism, Tyrannosauridae

## Abstract

Among extant species, the ability to sample the extremes of body size—one of the most useful predictors of an individual's ecology—is highly unlikely. This improbability is further exaggerated when sampling the already incomplete fossil record. We quantify the likelihood of sampling the uppermost limits of body size in the fossil record using *Tyrannosaurus rex* Osborn, 1905 as a model, selected for its comparatively well‐understood life history parameters. We computationally generate a population of 140 million *T. rex* (based on prior estimates), modelling variation about the growth curve both with and without sexual dimorphism (the former modelled after *Alligator mississippiensis*), and building in sampling limitations related to species survivorship and taphonomic bias, derived from fossil data. The 99th percentile of body mass in *T. rex* has likely already been sampled, but it will probably be millennia before much larger giants (99.99th percentile) are sampled at present collecting rates. Biomechanical and ecological limitations notwithstanding, we estimate that the absolute largest *T. rex* may have been 70% more massive than the currently largest known specimen (~15,000 vs. ~8800 kg). Body size comparisons of fossil species should be based on ontogenetically controlled statistical parameters, rather than simply comparing the largest known individuals whose recovery is highly subject to sampling intensity.

## Graphical abstract

Body size is central focus of evolutionary ecology, but it can be difficult to estimate in fossil organisms, particularly at the extreme limits of size variation. Here, we use population modelling to estimate maximum body size of the familiar *Tyrannosaurus rex*. We estimate that the largest ever *T. rex* may have been up to 70% larger than the current largest known specimen, and we explore the implications this might have had for the ecology and biomechanics of the animal.

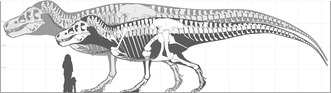

## 1 INTRODUCTION

Dinosaurs are the largest animals ever to have walked the earth. Accordingly, they inspire awe among the public, and frequently the question is asked: ‘Which is the biggest?’. But which is the biggest dinosaur (or example of some subclade or ecological guild) ever *found* and which is the biggest that ever *lived* are two separate considerations. The first is relatively straightforward to answer, notwithstanding complications in estimating body mass (Campione & Evans, 2020) and comparing skeletons of variable completeness (Persons et al., 2020). The second consideration is the more difficult to address. After all, to use a familiar example, ~2.5 billion (± an order of magnitude) individual *Tyrannosaurus rex* Osborn, 1905, are estimated to have existed over the course of the ~2.4 million‐year span of the species (Griebeler, 2023; Marshall et al., 2021), and, of these, only 84 reasonably complete skeletons—that is, those that are diagnostic to species level—have been collected (this number includes privately owned specimens; P. J. Currie, pers. comm. to JCM, 2022). Surely, having sampled just 3.4 × 10^−6^ per cent of the presumed total population, it is extraordinarily unlikely that palaeontologists have discovered the largest *T. rex* ever to have prowled the late Maastrichtian floodplains of North America. Such a scenario would require both that the record‐breaking individual was fossilized and that palaeontologists had the good fortune to discover it. Even so, knowing something about the upper limits of body size in a fossil species is interesting for a variety of reasons. For one, it may be informative about the total niche space occupied by that species (Peters, 1983). For another, if body size increase within a lineage is driven by directional selection, following some formulations of Cope's Rule (Sanisidro et al., 2023), then it would be useful to be able to consider the upper tail of the body size distribution on which natural selection is purported to have acted. Similar considerations would be informative concerning the absolute limits of large body size.

In this contribution, we attempt to address the likelihood that we have sampled the largest examples (i.e., within the uppermost percentiles of body mass) of some fossil species, using *T. rex* as a model. We also attempt to statistically derive an approximate maximum body mass estimate for the species. Beyond simply knowing ‘which is biggest?’, our goal here is to provide some context about how patchy sampling can affect our estimation of maximum body size in fossil species.

## 2 METHODS AND RESULTS

We will model a population of *Tyrannosaurus rex* to better understand the estimation and effects of sampling the upper limits of body size. Other dinosaur species (e.g., *Coelophysis bauri* [Cope, 1887], *Microraptor* Xu, Zhou & Wang, 2000 spp., *Psittacosaurus* Osborn, 1923 spp.) are known from more abundant and complete material, but their life history parameters have not been sufficiently reconstructed, and the species are therefore not amenable to our approach. *Tyrannosaurus rex* is also an interesting model organism because it already appears to have approached the upper limits of body size for a terrestrial biped.

We begin by considering somatic growth in *T. rex*. There are many ways to measure body size in a dinosaur (hip height, total body length, etc.) but we focus here on body mass because it scales closely (*R* ^2^ > .90) with femur circumference in extant bipedal tetrapods (Anderson et al., 1985; Campione et al., 2014) and is therefore easy to estimate. Femoral circumference scaling also has the benefit of requiring only a single bone for estimation—as opposed to a complete body fossil, which is exceedingly rare—and sample size is therefore increased accordingly. In any case, other methods for estimating body mass (e.g., volumetric‐density approaches) offer broadly congruent results that overlap in their prediction intervals (Campione & Evans, 2020). In this study, we use the scaling equation of Campione et al. (2014) to estimate body mass, given as:

(1) $$\log_{10}{\text{body mass}}_{\left(g\right)}=2.754\times \log_{10}{\text{femur circumference}}_{\left(\mathrm{mm}\right)}-0.683.$$

**FIGURE 1:**
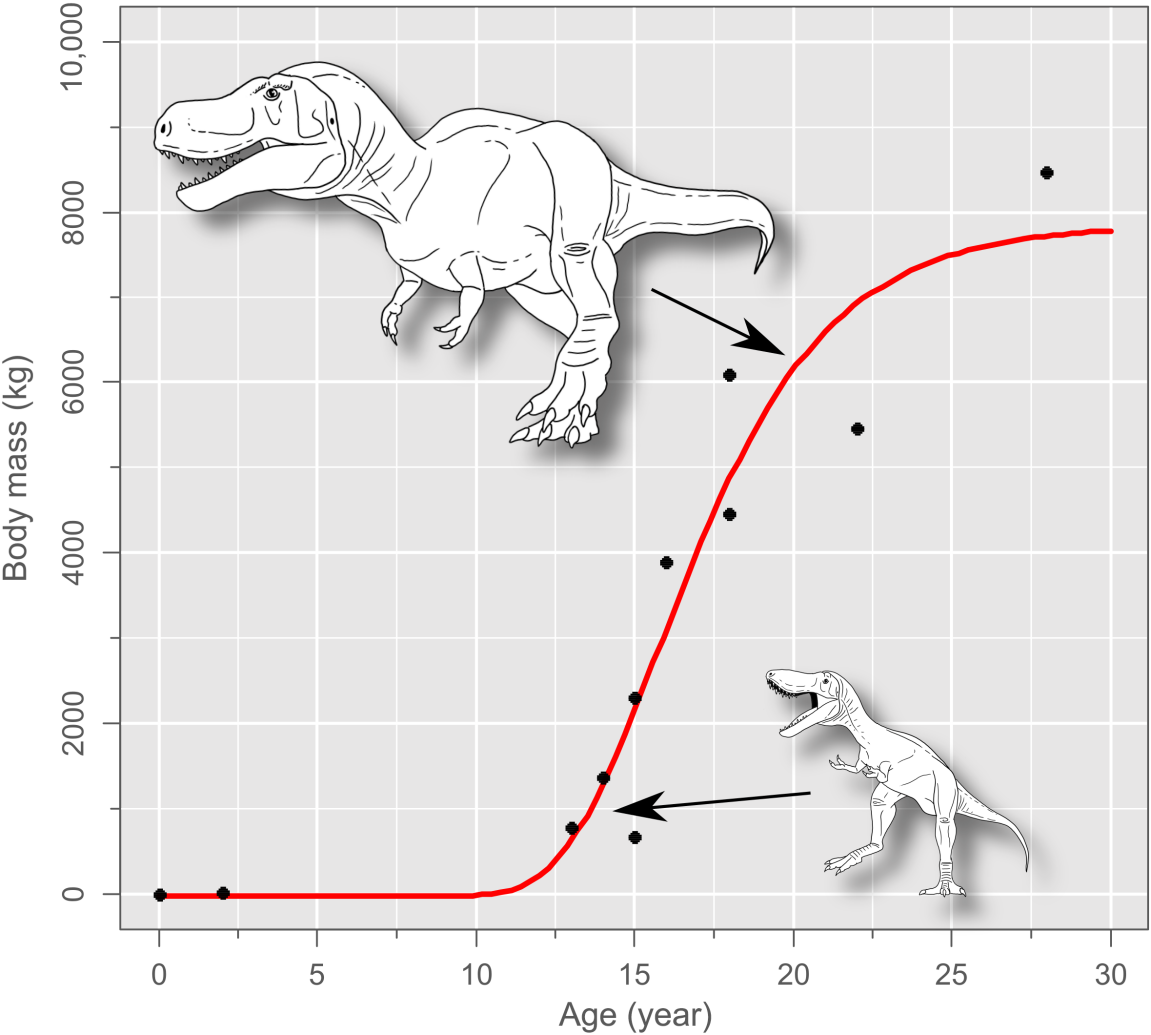
Four‐parameter Gompertz growth curve for *Tyrannosaurus rex*. Mathematical function given in Equation 2. Illustrations not to scale.

The details of somatic growth in *T. rex* were first elucidated by Erickson et al. (2004). They used a logistic growth model to describe body mass increase in the species with age (inferred using osteochronology), then updated the model (Erickson et al., 2016) following criticism from Myhrvold (2013). Subsequently, new age and body mass estimates have been published for various fossil individuals (e.g., Persons et al., 2020; Woodward et al., 2020), and we will use these new data and our own observations to model an updated growth curve for *T. rex.* (Note that we assume a body mass of 2 kg at hatching following Lee, 2016.) Our model (Figure 1; Equation 2) differs from that of Erickson et al. (2016) in that it is fitted with a four‐parameter Gompertz function, commonly used to fit growth data (Tjørve & Tjørve, 2017) (see Table S2 for other fitted growth models and corrected Akaike Information Criterion values used for model selection). The estimated asymptotic body mass is considerably higher than that given by Erickson et al. (2016) (7852 vs. 5649 kg), almost certainly due to our use of the femoral circumference scaling method of Campione et al. (2014) (Erickson et al., 2004 relied on the earlier method of Anderson et al., 1985, which also uses femoral circumference scaling and tends to yield lower mass estimates). The estimated instantaneous growth rate ($=\left[\text{growth rate}/4\right]\times \text{asymptote}$) is somewhat lower than given by Erickson et al. (2016) (652 vs. 774 kg/year). Our growth model is otherwise very similar in predicting negligible growth in the first decade of life (but see Section 3), followed by explosive growth during the teenage years, which is thought to have led to the impressive size attained by *T. rex* over other tyrannosaurids (Erickson et al., 2004). Maximum longevity is conservatively estimated to have been 30 years, based on the oldest known individuals, but future discoveries may extend the estimated longevity.

(2) $${\text{Body mass}}_{\left(\mathrm{kg}\right)}=7851.74-19.4308\cdot e^{e^{-0.332391\left({\mathrm{age}}_{\left(\text{years}\right)}-15.7611\right)}}.$$

We must now determine how large a population to model. Marshall et al. (2021) estimated a median total abundance of 2.5 billion individual *T. rex* that ever lived. (Griebeler, 2023 recently downgraded this estimate somewhat, but given the range of values we will be working with, her revised estimates are effectively bracketed here.) However, for the sake of working with a computationally more tractable number, we will assume a total abundance of 140 million, which is the lower median estimate provided by Marshall et al. (2021). We can thus generate a series of 140 million random ages, ranging between 0 and 30 years, along the growth curve in Equation 2.

Populations of *T. rex* did not exhibit an even age distribution; like other dinosaurs for which demographic data are available (Erickson, 2014), *T. rex* is estimated to have exhibited a Type B_1_ survivorship curve (Erickson et al., 2006), characterized by high neonate mortality that plateaus after the first year and increases again near senescence. This curve was calculated using data derived from osteohistology (reported previously in Erickson et al., 2004). Thus, at any one time, there is likely to have been much more skeletally and somatically immature *T. rex* on the Late Cretaceous landscape compared to large adults (Schroeder et al., 2021), a pattern that we will build into our population model by resampling our randomly generated ages (with replacement) according to the survivorship probability for *T. rex* given in Erickson et al. (2006):

$$n_{\text{survivors}}=n_{{\text{survivors}}_{\left(\mathrm{age}=0\right)}}\cdot e^{\left(\frac{0.002}{0.2214}\right)\left(1-e^{\left(0.2215\cdot {\mathrm{age}}_{\left(\text{years}\right)}\right)}\right)}$$

Next, it is necessary to associate these survivorship‐corrected age values with modelled population variation (residual error) in body mass about the curve. In most reptiles, body mass variation increases with age for three main reasons: (1) at the lower end of the growth curve (among neonates), body size variation is constrained by egg size; (2) genetic factors, influenced by stabilizing selection, can lead to increased intraspecific variation with sexual or somatic maturity (e.g., sexual dimorphism); (3) differential development in response to environmental variation (e.g., food, seasonality) ensures that accumulated differences become magnified over the course of development, particularly in species exhibiting multi‐year growth. Our sample (*n* = 11) is too small to reasonably estimate the true residual standard deviation of the original *T. rex* population. We must therefore impute variation about the growth curve using data from an extant model. A ratite model might be tempting, given the bipedal locomotion shared with *T. rex*, and the close phylogenetic relationship between non‐avian dinosaurs and birds. However, we argue that such a model is inappropriate because (1) ratite diets consist primarily of plants, disanalogous to the carnivory exhibited by *T. rex*; (2) the best‐studied ratites (ostriches and emus) typically live in seasonally dry grassland habitats, unlike the lowland, subtropical settings frequented by *T. rex* (Russell, 1989); and (3) whereas *T. rex* took decades to reach somatic maturity (Figure 1), ratites reach their maximum body size in just a single year (Cilliers et al., 1995; du Preez et al., 1992). This last point is crucial, because body size variation will be more strongly expressed in those species whose somatic terminus is subject to the vicissitudes of multi‐year variation in resource availability, which ratites are not; it is important to compare species with similarly shaped growth curves (cf. Hone & Mallon, 2017). Moreover, the demographic data (e.g., global population size, body mass variance) necessary to produce a reliable model of wild ratite growth variance simply are not yet available. For these reasons, we will choose the American alligator (*Alligator mississippiensis* Daudin, 1801), which is a much better analogue in the above regards (e.g., Grigg & Kirshner, 2015; Rootes et al., 1991; Wilkinson et al., 2016), and which has more comprehensive population demographics. Crocodylians are also the next‐most closely related group to dinosaurs, and include the largest living reptiles. However, unlike *T. rex*, which is not demonstrably sexually dimorphic in body size (Mallon, 2017; Saitta et al., 2020), possibly owing to sampling limitations (Hone & Mallon, 2017), *A. mississippiensis* exhibits strong sexual size dimorphism; on average, somatically mature males weigh nearly three times more than females (Wilkinson et al., 2016). Using *A. mississippiensis* as a model consequently has the added benefit of bracketing the upper limits of size variance in our calculations.

We will therefore allow the standard deviation to vary between 0 kg (no body size variation) at the lower end of the growth curve and one of two values at the upper end (asymptote), each modelled on the statistical dataset of Wilkinson et al. (2016) for a wild population of *A. mississippiensis* from the Tom Yawkey Wildlife Center in South Carolina, near the northern range limit of the species. (Note that the size data of Wilkinson et al., 2016 are given as snout‐vent lengths, which we convert to body mass using the allometric scaling relationship given in appendix table 3 of Hurlburt et al., 2003; see Table S3 and S4). First, assuming no sexual dimorphism in *T. rex*, we pool the standard deviations of somatically mature male and female *A. mississippiensis* (the former varying more widely) and scale up the resulting value to the asymptotic body mass calculated for *T. rex* in Equation 2. This yields a scaled standard deviation of 1226 kg and a lower maximum body mass estimate for *T. rex* (Table S7). Second, assuming strong sexual size dimorphism in *T. rex* equivalent to that observed in *A. mississippiensis*, we scale up the male–female asymptotic offset of *A. mississippiensis* to the *T. rex* asymptote given in Equation 2 and scale up the associated standard deviations about the male–female growth curves accordingly. This yields a scaled standard deviation of 2243 and 735 kg for modelled male and female *T. rex*, respectively, and an upper estimate for the maximum body mass attained by *T. rex*. In both scenarios (with and without sexual dimorphism), we fit a logistic function to the scaling of the standard deviation (a reasonable but uncorroborated assumption) to yield the following:

$$\sigma_{\left(\mathrm{no}\;\text{dimorphism}\right)}=1225.776+\left(\frac{-1225.776}{1+\left[\frac{{\mathrm{age}}_{\left(\text{years}\right)}}{612.8881}\right]}\right)$$

$$\sigma_{\left(\text{males}\right)}=2242.966+\left(\frac{-2242.966}{1+\left[\frac{{\mathrm{age}}_{\left(\text{years}\right)}}{1121.483}\right]}\right)$$

$$\sigma_{\left(\text{females}\right)}=735.0143+\left(\frac{-735.0143}{1+\left[\frac{{\mathrm{age}}_{\left(\text{years}\right)}}{367.5071}\right]}\right)$$

**FIGURE 2:**
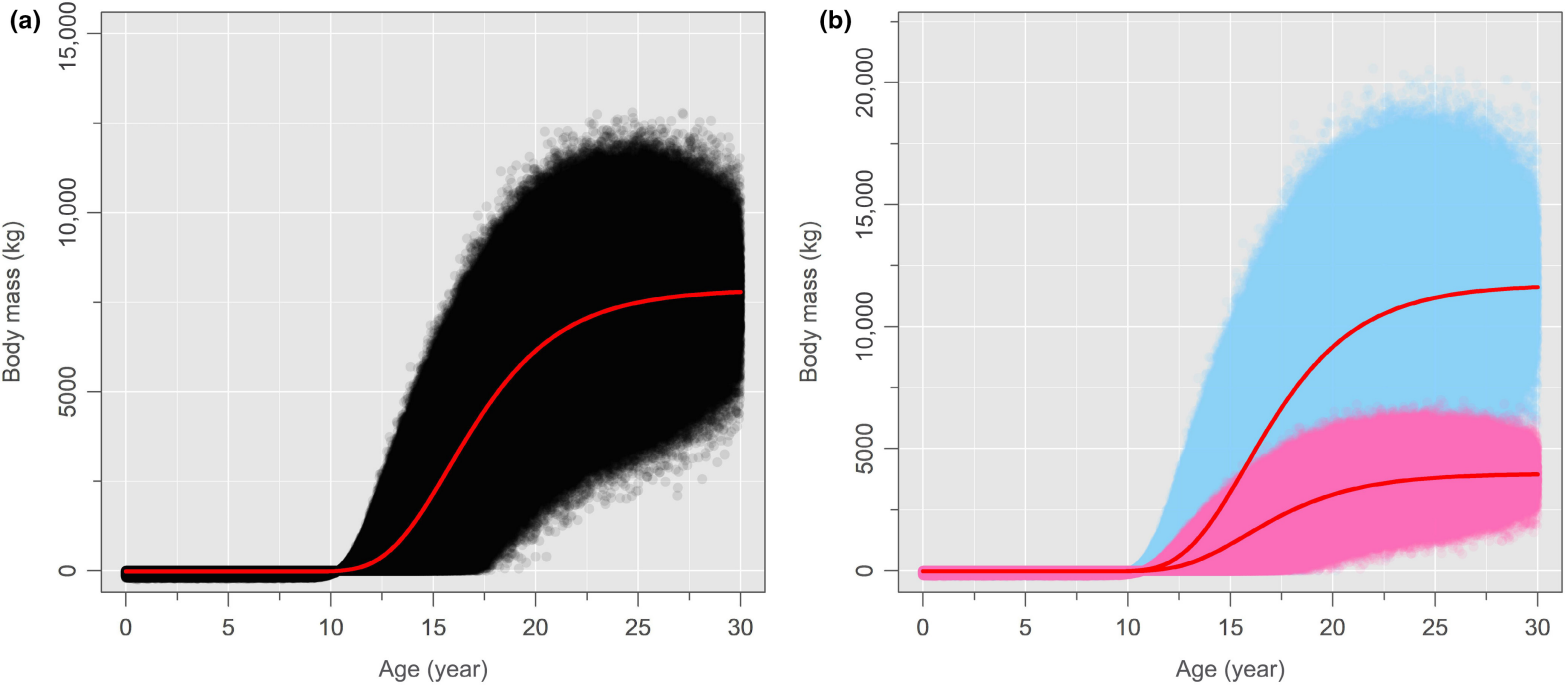
*Tyrannosaurus rex* total population models (*N* = 140 million individuals) assuming (a) no sexual dimorphism and (b) sexual dimorphism (modelled after *Alligator mississippiensis*). Note that the scaling of the y‐axis varies between (a) and (b). In (b), blue corresponds to the larger males and pink to the smaller females, following the pattern of sexual dimorphism expressed in *A. mississippiensis*, although the sexual differences were possibly reversed in *T. rex*. Each model implements survivorship data for *T. rex*, seen as the drop‐off in abundance prior to death at 30 years.

**FIGURE 3:**
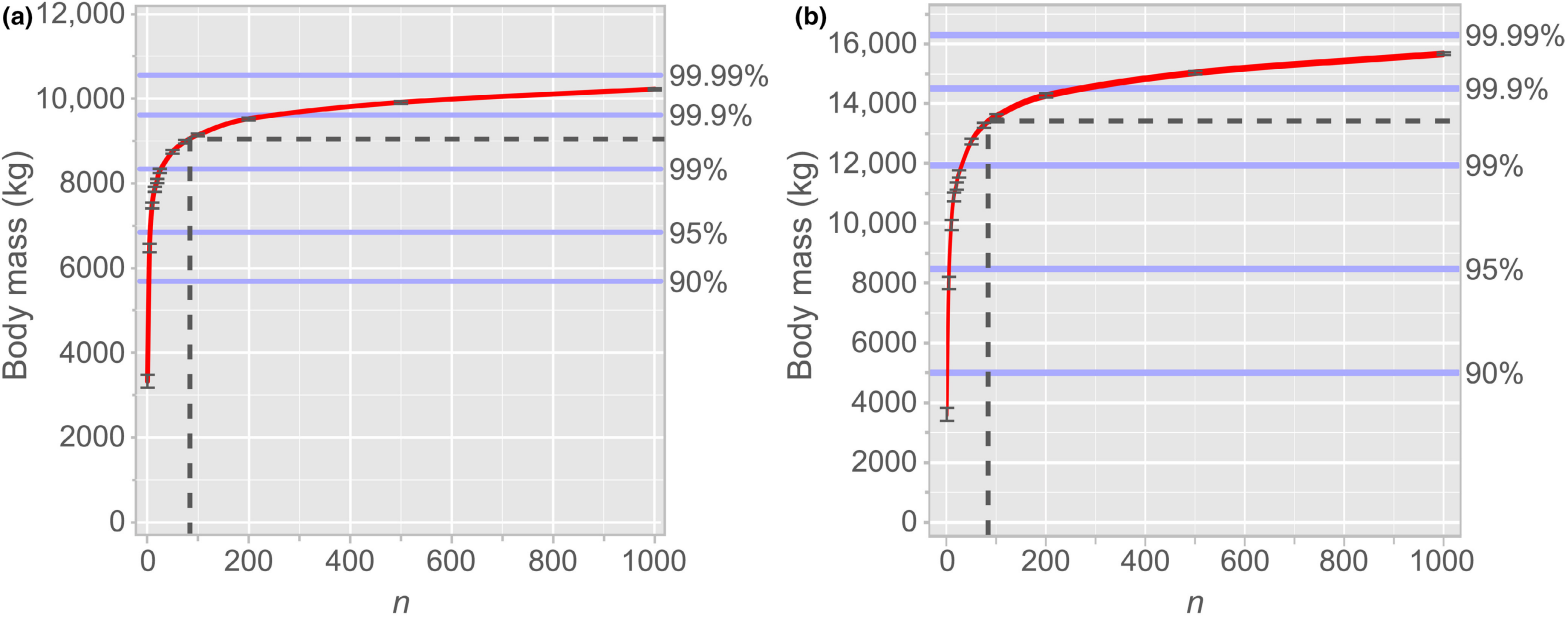
Sampling effort required to achieve uppermost body size percentiles for *Tyrannosaurus rex*. (a) Assuming no sexual dimorphism; (b) assuming sexual dimorphism (cf. *Alligator mississippiensis*). Ninety‐five per cent confidence interval shown for each modelled level of sampling effort. Dashed line indicates present sampling effort (*n* = 84).

We have thus modelled a standing population of 140 million *T. rex*, assuming sexual dimorphism and otherwise (Figure 2), which we must now sample to determine how sampling effort affects the likelihood of recovering the largest body sizes (90th, 95th, 99th, 99.9th, and 99.99th percentiles). We will therefore sample the modelled population 1000 times (without replacement) for predetermined levels of sampling effort (*n* = 1, =5, =10, =15, =20, =25, =50, =75, =100, =200, =500, and =1000). However, the nature of the vertebrate fossil record is such that not all body sizes are preserved or sampled equally. Within the Upper Cretaceous fluvial deposits of North America, vertebrate body sizes of less than 60–70 kg are underrepresented, due to taphonomic and collector bias (Brown et al., 2013, 2022). Indeed, to date, examples of neonate tyrannosaurids are exceedingly rare and fragmentary (Funston et al., 2021). We will thus build this size bias into our model by sampling only those *T. rex* individuals weighing >70 kg, in accordance with Brown et al. (2022). The sampling results are given in Figure 3 (associated data in Tables S8 and S14). Further methodological details and calculations are provided in Appendix S1. R scripts are provided in Appendix S2 (no sexual dimorphism model) and Appendix S3 (sexual dimorphism model).

## 3 DISCUSSION

### 3.1 Sampling complications (past and present)

Despite a bevy of popular and technical literature asking, ‘which is the biggest?’, meaningful comparisons of body size in the fossil record—particularly at the uppermost extremes—are complicated (Persons et al., 2020). Taphonomic and collector biases ensure that skeletons are rarely complete, minimizing overlap of certain critical body parts for comparison (e.g., skull vs. femur vs. vertebral column). Yet even well‐preserved fossils, represented by reasonably large sample sizes, may suffer from biases of their own. Extant models show that body size variation can occur between populations (e.g., *Crotalus viridis* Rafinesque, 1818: Ashton, 2001), including between islands (e.g., *Varanus komodoensis* Ouwens, 1912: Jessop et al., 2006), and may result from variation in levels of sexual dimorphism (e.g., *Morelia spilota imbricata* [Smith, 1981]: Pearson et al., 2002). Even body size within a single population may vary through time in response to selective evolutionary pressures (e.g., *Lynx canadensis* Kerr, 1792: Yom‐Tov et al., 2007), and individuals may vary widely in body mass not just within their lifetimes but seasonally (over 35% in *Ursus americanus* Pallas, 1780: Hellgren, 1998). These additional sources of variation conspire to ensure that, unless population sampling is both intensive and spatiotemporally exhaustive, it can be difficult to establish the upper limits of body size even for extant species, considerations of which typically do not even incorporate historical population data.

By way of a more practical example, Woodward et al. (1995) noted that, of the 72,760 *Alligator mississippiensis* individuals harvested in Florida between 1977 and 1994, just 0.01% were >4 m in total length (the largest confirmed record at the time was 4.27 m long, harvested in 1989). But despite this intensive sampling, several larger individuals have been recorded outside of Florida, in Texas and Alabama (Brunell et al., 2013). The largest confirmed *A. mississippiensis* was harvested in Alabama in 2014 and measured 4.50 m long (Brunell et al., 2015). Given that it has been less than a decade since the last known record length was reported, and considering that the total wild population of *A. mississippiensis* is estimated to be somewhere between 3 and 4 million individuals (Elsey & Woodward, 2019), it is not unreasonable to suspect that considerably larger animals might still be found. Indeed, assuming the above total wild population figures, the statistical data for senescent *A. mississipiensis* in Table S3, and an adult population composition of just 5% (Eversole et al., 2018), *z*‐score analysis suggests that the very largest living individuals might approach 5.0–5.1 m in length (Table S15). These estimates comport with some uncorroborated reports of individuals >5 m long (Woodward et al., 1995), though certainly not the most extreme among them (e.g., McIlhenny, 1935 reported several unverifiable specimens that were supposedly >5.2 m long).

The question, therefore, remains: what is the likelihood that we have sampled even a very large individual of any fossil species, most of which are known from few and incomplete remains? The question is not merely academic; dinosaurs included the largest animals ever to have walked the earth, so understanding how our limited sampling of the fossil record affects our ability to recover the upper limits of body size will surely bear on considerations about our capacity to study the limitations of body size in terrestrial animals and the possible range of ecologies exhibited by the exceptionally large species (Schmidt‐Nielsen, 1984).

### 3.2 Likelihood of sampling the largest *T. rex*

Our results indicate that, given present sampling efforts (*n* = 84), the likelihood that we have sampled even the 99th percentile of body mass for *Tyrannosaurus rex* is quite good (Figure 3). These numbers, however, are contingent on our modelling; the degree to which it reflects reality is uncertain. Although our growth curve (Figure 1, Equation 2) does not differ substantially from previously published estimates, we concede that it is very likely inaccurate in that it posits near‐zero growth for the first decade of life (as does the growth curve of Erickson et al., 2004, 2016). New fossil finds and the broader application of osteohistological methods will surely clarify early growth in *T. rex*, but given that our study concerns the upper limits of body size in this taxon, this presumed inaccuracy has comparatively little bearing on our analysis of sampling effort. (Longrich & Saitta, 2024 recently published a growth curve for *T. rex* that posits exponential growth in the first decade of life, based on back estimation of line of arrested growth counts. However, asymptotic body mass remains very similar to that used here.) The same is true of certain other of our modelling parameters, including the presence of sexual dimorphism (Figure 3), survivorship, and minimum taphonomic size bias (to a point). Variation in total abundance has the greatest effect on sampling effort, for the greater the size of the population, the greater the sampling effort will be required to recover the largest individuals. To this end, if the original total abundance of *T. rex* was closer to the median estimate of 2.5 billion of Marshall et al. (2021), the likelihood of our having sampled the uppermost percentiles will be correspondingly diminished. According to our model, and assuming a linear rate of discovery of 0.694 skeletons/year (based on the above specimen count for the years 1900–2021), it may be another quarter century before we are likely to sample the truly gigantic (99.9th percentile) *T. rex*, and millennia before we ever sample the largest individuals of all (>99.99th percentile).

### 3.3 How big could *T. rex* get?

**FIGURE 4:**
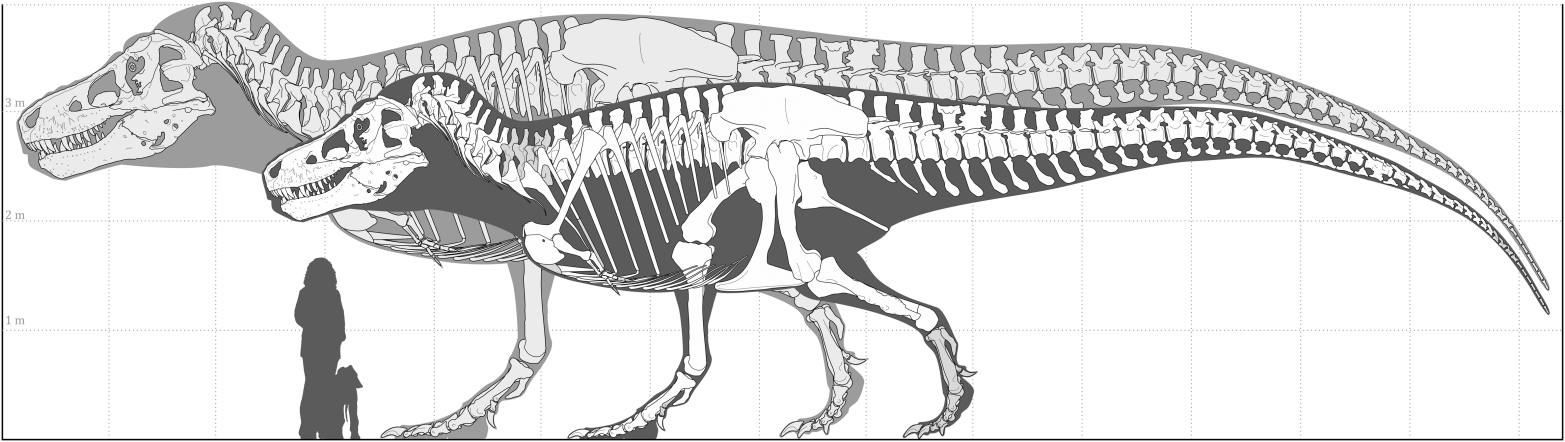
Comparison of FMNH PR 2081 (‘Sue’), among the world's largest known *Tyrannosaurus rex*, to the estimated largest possible *T. rex* in the background. Human with dog silhouette for scale. Note: the larger skeleton has been scaled up from the smaller according to allometric principles. Reconstruction © Mark Witton (used with permission).

Given uncertainties about body mass estimation, growth curve estimation, variation about the growth curve, and population modelling, this is the more difficult question to answer. The largest individuals generated by our population modelling (*N* = 140 million) are 13,026 ± 3257 kg (mean prediction error from Campione et al., 2014) (assuming no dimorphism) and 21,465 ± 5366 kg (assuming dimorphism). For reasons discussed below (Section 3.4), the latter estimate seems highly unlikely, so estimation of maximum body size under the strong sexual dimorphism scenario (cf. *Alligator mississippiensis*) will not be considered further. Judging by the growth curve in Figure 1, body mass in *Tyrannosaurus rex* asymptotes near 25 years of age; individuals older than this account for ~0.8% of the total population. As in the alligator example given earlier, we can apply this proportion, along with the statistical data for senescent *T. rex* in Table S7, to a *z*‐score analysis (Table S16) assuming a total population of 2.5 billion *T. rex* individuals (Marshall et al., 2021). This yields body mass estimates at +3 standard deviations of >11,000 ± 2750 kg, and an absolute maximum body mass for *T. rex* of ~15,000 ± 3750 kg, which is ~70% larger than the mass estimate for RSM P2523.8 (‘Scotty’), the currently largest known *T. rex* at ~8870 kg (Persons et al., 2020). We think it likely, therefore, that substantially larger *T. rex* than those presently known must once have existed, but it is extremely unlikely that they are preserved in the fossil record, given their infrequency. Retrocalculating from Equation 1 above, we estimate that an individual weighing 15,000 kg would have a minimum femur circumference of ~715 mm (compared to 590 mm in RSM P2523.8). Comparable femur circumferences occur only in sauropods (Benson et al., 2018). By estimating femur length from body mass using the theropod scaling equation of Christiansen and Fariña (2004), and subsequently estimating total body length from femur length using the tyrannosaurid scaling equation of Currie (2000), a 15,000 kg individual *T. rex* would likely have exceeded 15 m in length (Figure 4; Table S17). For comparison, ‘Sue’ (FMNH PR 2081), one of the largest and most complete *T. rex*, is ~12 m long. We stress that our maximum mass estimate of ~15,000 kg is a conservative one; if sexual size dimorphism in *T. rex* were more strongly expressed (although not so strongly as in *A. mississippiensis*), the size variability among the adults would increase accordingly, as would the maximum size estimate.

How does our maximum size estimate compare to the maximum theoretical size attainable for a bipedal carnivore? Published estimates for such animals are rare and tend to focus instead on quadrupedal herbivores, which could reach much larger body sizes (e.g., Alexander, 1998; Economos, 1981; Fortelius, 1993; Hokkanen, 1986). Paul (1997:135) opined, "The largest unpreserved world record individual predatory dinosaurs probably approached 15 tonnes", which remarkably agrees with our maximum estimate for *T. rex*, but he unfortunately provided no information about how he reached such a number. Many factors limit body size within terrestrial vertebrates, including bone strength (Farlow et al., 1995), movement requirements (Henderson, 2023), ecological energetics, minimum viable population size (Farlow, 1993), thermal regulation (Spotilla et al., 1991), and available evolutionary time (Burness et al., 2001), among others. However, few of the studies just cited provide an estimate for the maximum body size at which these limitations would have been prohibitive for a large theropod. Henderson (2023) suggested that theropods in excess of 12 m long would have had greatly reduced acceleration capacity and therefore were at a disadvantage when it came to prey capture and evasion. But perhaps these issues might not have been as prohibitive for a hypothetical 15 m individual as feared; Ruxton and Houston (2003) argued on the grounds of ecological energetics that scavenging was a viable option for *T. rex* (but see Carbone et al., 2011 and Kane et al., 2016 for an opposing view), and it seems rather unlikely that a maximally sized individual had much need to evade anything. Ultimately, provided ideal environmental conditions (which vary considerably and are ultimately difficult to estimate), it seems likely that bone strength would dictate the uppermost theoretical limits on body size. Using the limb bone scaling equation of Campione and Evans (2012) for quadrupeds, Benson et al. (2018) calculated the body mass of the Chicago skeleton of *Brachiosaurus altithorax* Riggs, 1903 (FMNH P 25107; the largest specimen for which such an estimate could be produced, although other lesser‐known sauropods were probably larger) at ~56,000 kg. Assuming (reasonably) that the species was capable of only a slow walk, with a single limb suspended mid‐air at a time, and assuming that the body mass loaded equally across the remaining limbs (which minimizes the weight borne by any single one), this gait amounts to an average mass of ~18,000 kg borne per limb. This figure is considerably higher than our estimated maximum body mass for *T. rex*, although it seems likely that a 15,000 kg biped would be flirting with the same limits of bone scaling as a large sauropod. More work along these lines is clearly required. Information pertaining to the allometry of cortical bone thickness in *T. rex* femora would facilitate the estimation of bone strength (Farlow, 1995) and better inform the structural feasibility of a maximally sized individual. Our 15,000 kg maximum body mass estimate for *T. rex* is not based on hard fossil data, and should be treated as a null hypothesis in the absence of size‐limiting constraints.

### 3.4 Further implications

Our model assuming strong sexual size dimorphism in *T. rex* (Figure 2b) appears to contradict the known distribution of body size in the species, and so offers an argument against such strong dimorphism having been present in reality. If, as demonstrated above, we have already sampled the upper percentiles (95th–99th) of body mass in *T. rex* (Figure 3), then we should observe both a higher variance in body mass among skeletally mature individuals and much larger individuals than currently known. None of this is to say that *T. rex* was not sexually dimorphic; the male–female body size disparity in *A. mississippiensis* is at the upper end of the sexual size dimorphism spectrum for reptiles (Fitch, 1981; Wilkinson & Rhodes, 1997) and so may not be a good model in this sense. Sexual size dimorphism may still have been present in *T. rex*, but at lower levels than assumed by the present study. Recent efforts have failed to statistically demonstrate sexual size dimorphism in *T. rex* (Mallon, 2017; Saitta et al., 2020), likely for issues related to poor sampling control and the confounding effects of multi‐year growth (Hone & Mallon, 2017).

Large dinosaurs in general (Varricchio, 2011; Wyenberg‐Henzler et al., 2022), and tyrannosaurids in particular (Holtz, 2021; Schroeder et al., 2021), likely occupied different ecological niches during ontogeny. In the case of *T. rex*, this ontogenetic niche shift was accompanied by changes in bite performance and locomotor characteristics (Hutchinson et al., 2011; Lautenschlager, 2022; Rowe & Snively, 2022; Therrien et al., 2021), among others. Although the implications are not immediately obvious, truly gigantic individuals that were twice the mass of currently known mature specimens would potentially have had different resource requirements and mechanical capabilities than more typical adults (cf. Hone et al., 2020). For example, Kane et al. (2016) used ecological energetic modelling to show that a hypothetical 15,000 kg *T. rex* would have required more than twice the daily caloric intake of an average‐sized (6000 kg) adult. From these perspectives, such enormous individuals would prove interesting in terms of the upper mechanical and ecological limits of functioning carnivores. Theropods (including tyrannosaurids) generally preferred prey smaller than themselves (Hone & Rauhut, 2010), but particularly immense individuals may have been able to access resources not otherwise available to more typically‐sized adults. Pods of killer whales (*Orcinus orca* Linnaeus, 1758) are known to feed at least occasionally on adult blue whales (*Balaenoptera musculus* [Linnaeus, 1758])—the largest vertebrates that ever lived (Totterdell et al., 2022). The considerations of Henderson (2023) notwithstanding, it is not inconceivable that very large *T. rex* could likewise have felled sympatric adult titanosaurs—the largest terrestrial vertebrates that ever lived—at least on occasion (Sampson & Loewen, 2005).

## 4 CONCLUSIONS

Given present sampling efforts, we have likely already sampled the 99th percentile of body mass in *Tyrannosaurus rex*. We estimate that the largest ever *T. rex* may have been up to 70% larger than the largest currently known, although the likelihood that it is preserved in the fossil record is infinitesimal. This figure is contingent on our modelling, which relies heavily on data from extant alligator populations. During peer review of this study, one reviewer insisted on the use of a corresponding ratite model for the sake of creating an extant phylogenetic bracket (sensu Witmer, 1995). We argued against this for the a priori reasons given in Section 2 above, and because comparable demographic data for wild ratite populations are yet unavailable. Regardless, our preference for the no‐dimorphism model of body size variance in *T. rex* is likely to bring our maximum body size estimate in closer agreement to one produced by a ratite model, rather than the highly implausible numbers produced by a strict adherence to the dimorphic alligator model.

In any case, the 70% value is not key to our argument; our central tenet is that, although many dinosaur lineages produced animals of incredible size, the very largest individuals would almost certainly have been considerably larger than those we know of presently. Somatically mature or senescent dinosaurs are, in fact, relatively rare findings (Horner et al., 2011; Hone et al., 2016; Woodward et al., 2015), so this claim should not be controversial. Even aside from issues associated with the estimation of somatic growth, population size, life history, and more, the likelihood that even a fossil species known from several hundred complete skeletons would encompass the very largest individuals of that species is vanishingly small. And yet the reality is that these parameters remain unknown for nearly all dinosaurs, which are represented by relatively few and scant remains. To the best of our knowledge, ours is the first attempt to navigate these difficulties to reach some reasonable maximum size estimate for a fossil species and complement prior mechanical, physiological, and ecological means of approximating the same. We stress that our maximum size estimate for *T. rex* is statistically derived, and does not explicitly factor in other practical concerns relating to structural or ecological limitations.

Although our findings may make for a nice hyperbolic headline (‘Dinosaurs even larger than currently known’), we would prefer to end on a more sobering note. There is, inevitably, great popular and scientific interest in the extremes of large body size. Our findings serve as an important reminder that body size comparisons of fossil species should entail ontogenetically controlled statistical parameters (e.g., means and variances). Simply comparing the largest known individuals ignores the realities of intraspecific variation and sampling intensity, and is more likely to lead to claims that are debatable, if not outright misleading. We maintain that the rigid pursuit to establish ‘which is biggest’ can distract from more interesting biological questions posed by the immensity of the non‐avian dinosaurs.

## AUTHOR CONTRIBUTIONS

**Jordan C. Mallon:** Conceptualization (equal); data curation (lead); formal analysis (lead); funding acquisition (lead); investigation (equal); methodology (lead); visualization (lead); writing – original draft (lead); writing – review and editing (equal). **David W. E. Hone:** Conceptualization (equal); data curation (supporting); formal analysis (supporting); investigation (supporting); methodology (supporting); writing – original draft (supporting); writing – review and editing (equal).

## FUNDING INFORMATION

Funding for this project was provided by the Canadian Museum of Nature.

## CONFLICT OF INTEREST STATEMENT

The authors declare no conflict of interest.

## Supporting information


Appendix S1.

Appendix S2.

Appendix S3.

## Data Availability

The data that supports the findings of this study are available in the supplementary material of this article.
