# Correction to “Estimation of maximum body size in fossil species: A case study using *Tyrannosaurus rex*”

**DOI:** 10.1002/ece3.70258

**Published:** 2024-09-04

Mallon JC, Hone DWE. Estimation of maximum body size in fossil species: A case study using *Tyrannosaurus rex*. *Ecol. Evol*. 2024;14: e11658. https://doi.org/10.1002/ece3.11658

The original version of this article used the wrong image in Figure 4. The correct image, included here, replaces an isometrically upscaled skeleton of the *Tyrannosaurus rex* specimen FMNH PR 2081 (‘Sue’) with an allometrically scaled skeleton. The original caption applies.

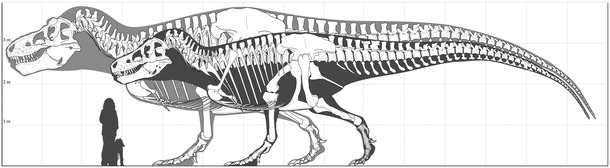

We apologize for this error.

