# Correction to “Estimation of Maximum Body Size in Fossil Species: A Case Study Using *Tyrannosaurus rex*”

**DOI:** 10.1002/ece3.73068

**Published:** 2026-02-03

Mallon, J. C., and D. W. E. Hone. 2024. “Estimation of Maximum Body Size in Fossil Species: A Case Study Using *Tyrannosaurus rex*.” *Ecology and Evolution* 14: e11658. https://doi.org/10.1002/ece3.11658.

We wish to acknowledge that the four‐parameter Gompertz growth model fitted to our data yields slightly negative body sizes below approximately ten years of age—a biological impossibility. This initially escaped our attention, because the large scale of the growth plot in Figure 1 obscures those comparatively minute negative values. We confirm that the lower asymptote (−19.4308 kg) is accurate to the model fitted by the Growth II software that we used (Table S2). Nevertheless, while we recognize (as we did in the originally study) that the lower end of the *Tyrannosaurus rex* growth curve is poorly reflective of life conditions, this does not affect our subsequent conclusions for two reasons: (1) All values < 70 kg were discarded anyway, because of the taphonomic filter we implemented; and (2) we were primarily interested in the upper end of the growth curve, which is better constrained by the data. Thus, we remain confident in our findings, insofar as they relate to our estimation of the upper limits of body size in *T. rex*.

We thank Dr. Nathan Myhrvold for bringing this issue to our attention and apologize for the error.

